# Zero-Fluoroscopy Cardiac Ablation: Technology Is Moving Forward in Complex Procedures—A Novel Workflow for Atrial Fibrillation

**DOI:** 10.3390/biology10121333

**Published:** 2021-12-15

**Authors:** Matteo Bertini, Graziella Pompei, Paolo Tolomeo, Michele Malagù, Alessio Fiorio, Cristina Balla, Francesco Vitali, Claudio Rapezzi

**Affiliations:** Cardiologic Unit, Translational Medicine Department, Sant’Anna University Hospital of Ferrara, 44124 Ferrara, Italy; pmpgzl@unife.it (G.P.); tlmpla@unife.it (P.T.); mlgmhl1@unife.it (M.M.); alessio.fiorio@edu.unife.it (A.F.); cristina.balla@unife.it (C.B.); vtlfnc@unife.it (F.V.); claudio.rapezzi@unife.it (C.R.)

**Keywords:** atrial fibrillation ablation, zero fluoroscopy, steerable sheath, intracardiac echo

## Abstract

**Simple Summary:**

Electrophysiological procedures are mainly performed using fluoroscopy, exposing both healthcare staff and patients to a non-negligible dose of radiation. To date, simple ablation procedures have often been approached with zero fluoroscopy. In complex ablation procedures, such as atrial fibrillation (AF) ablation, zero fluoroscopy is still challenging mainly because of transseptal puncture. We report a workflow to perform a complete zero-fluoroscopy AF ablation using a 3D electro-anatomical mapping system, intracardiac echocardiography and a novel steerable guiding sheath visible on the mapping system. We describe two cases, one with paroxysmal AF and the other with persistent AF during which this novel workflow was successfully applied with complete zero-fluoroscopy exposure and achieving pulmonary vein isolation.

**Abstract:**

Background and Rationale. A fluoroscopy-based approach to an electrophysiological procedure is widely validated and has been recognized as the gold standard for a long time. The use of fluoroscopy exposes both the healthcare staff and the patient to a non-negligible dose of radiation. To minimize the risks associated with the use of fluoroscopy, it would be reasonable to perform ablation procedures with zero fluoroscopy. This approach is widely used in simple ablation procedures, but not in complex procedures. In atrial fibrillation (AF) ablation procedures, fluoroscopy remains the main technology used, in particular to guide the transseptal puncture. Main results and Implications. We present a workflow to perform a complete zero-fluoroscopy ablation for AF ablation procedures using a 3D electro-anatomical mapping system, intracardiac echocardiography and a novel steerable guiding sheath that can be visualized on the mapping system. We present two cases, one with paroxysmal AF and the other one with persistent AF during which we applied this novel workflow achieving a successful pulmonary vein isolation without complications and complete zero-fluoroscopy exposure.

## 1. State of the Art

Over the last three decades, interventional cardiology has made great progress in the field of electrophysiology, particularly concerning the development of advanced techniques in electrophysiological studies and ablation procedures. Fluoroscopy-based approaches are widely validated and have been recognized as the gold standard for a long time. The use of fluoroscopy exposes both the healthcare staff and the patient to a non-negligible dose of radiation. For example, a procedure of atrial fibrillation (AF) ablation exposes patients to a dose up to 60 mSv and raises the absolute lifetime risk of a fatal cancer in an adult by 0.08% [1]. Furthermore, the latest evidence shows that the median per year radiation exposure for interventional cardiologists is up to three times higher compared with a typical radiologist [2]. To minimize the risks, it would be reasonable to take steps to comply with the ALARA (as low as reasonably achievable) principles, as established by an expert consensus document drawn up by the American College of Cardiology in 1998 [3]. Advanced 3D electro-anatomic mapping (EAM) systems have transformed catheter-based ablation, allowing the electrophysiologist a new insight into the study of complex arrhythmia, such as AF and ventricular tachycardia. All mapping systems, even if based on different principles, allow a precise localization of ablation catheter from the vascular access to the heart chambers. While scanning different cardiac structures with the catheter, the system records data about catheter localization and signals information. Spatial and electrical data are used together by the system to create an accurate reproduction of the 3D geometry of cardiac structures. Remarkably, using 3D EAM systems does not necessarily mean less radiation for patients and medical staff. A multicenter prospective study showed that reduction of fluoroscopy time is associated with patient’s age, type of arrhythmia, and operator’s expertise [4]. Despite the meaningful improvements in the field of arrhythmia mapping and ablations, ventricular tachycardia and AF still represent a great challenge for electrophysiologists. Although these arrhythmias are characterized by a greater complexity in terms of procedural maneuvers and activation mapping, newer mapping systems proved to be safe and effective approaches even for these arrhythmias [5]. Recently, catheter ablation has become increasingly adopted for symptomatic AF. According to the European society of cardiology guidelines, pulmonary vein isolation (PVI) should/may be considered as a first-line rhythm control therapy to improve symptoms in selected patients with symptomatic paroxysmal AF episodes (IIa), or persistent AF without major risk factors for AF recurrence as an alternative to antiarrhythmic drug class I or III, considering patient choice, benefit, and risk (IIb) [6]. Considering the worldwide spreading of this technique and the high exposure of radiation related to transseptal puncture and pulmonary vein isolation, promoting zero-fluoroscopy strategies in AF ablation is crucial to be in line with ALARA principles [4,7,8]. Nowadays, the use of fluoroscopy in radiofrequency ablation of AF is markedly reduced as compared to few years ago [9]. AF ablation procedures are often performed with low doses of fluoroscopy, but procedures with complete zero fluoroscopy are anecdotic. A workflow to reduce radiation use was recently published with satisfactory results, but this approach includes the use of transesophageal echocardiography guidance for the transseptal puncture with the need of cardiologists experienced in cardiac imaging during the ablation procedure [10]. A complete zero-fluoroscopy approach performed by electrophysiologists is possible using intracardiac echocardiography (ICE), but it is only limited and no standardized data are present [11]. Therefore, most of the procedures are still performed with low fluoroscopy use, mainly because transseptal maneuver is still performed under fluoroscopic guidance [12]. A complete zero-fluoroscopy approach using the combination of ICE and visualization of transseptal sheath through 3D EAM has not been systematically described. We report our initial experience in radiofrequency AF ablation without the use of fluoroscopy (complete zero fluoroscopy), describing our workflow for both paroxysmal and persistent AF procedures. To achieve zero fluoroscopy, we use the Carto 3^®^ EAM system (Biosense Webster, Johnson & Johnson Medical S.p.a., Irvine, CA, USA), ICE with CartoSound^®^ module (Biosense Webster, Johnson & Johnson Medical S.p.a., Irvine, CA, USA), and the VIZIGO™ steerable sheath (Biosense Webster, Johnson & Johnson Medical S.p.a., Irvine, CA, USA), which can be visualized on the mapping system.

## 2. Case 1: Paroxysmal Atrial Fibrillation

The first patient is a 53-year-old male with a long history of paroxysmal atrial fibrillation. He had some cardiovascular risk factors, including high blood pressure that had been well controlled for 5 years on a therapy with ACE inhibitor. He did not suffer from other relevant comorbidities, except for sleep apnea syndrome on home nocturnal ventilatory support. He had his first episode of paroxysmal atrial fibrillation six years before and he started oral anticoagulation with dabigatran (CHA2DS2-VASC score 1) and antiarrhythmic therapy, initially with Flecainide and then with Amiodarone. Despite this, he had been admitted several times to the emergency room due to irregular heartbeat episodes and other symptoms, such as palpitations and shortness of breath. The arrhythmic recurrences often required electrical or pharmacological cardioversions to control the symptoms. The patient was then referred to our clinic because of the gradual worsening of his symptoms. The arrhythmic episodes lasted several hours, with spontaneous resolution, crippling him in his daily life. Considering his young age, the absence of cardiac structural alterations, and the inefficacy of the rhythm control strategy, AF ablation was proposed to the patient. At admission into the ward, the patient was overweight (body mass index was 38 kg/m^2^) and the electrocardiogram showed a normal sinus rhythm, while the pre-procedure echocardiography revealed dilated left atrium (left atrial volume 43 mL/m^2^) and the ejection fraction of the left ventricle of 55%.

**Figure 1:**
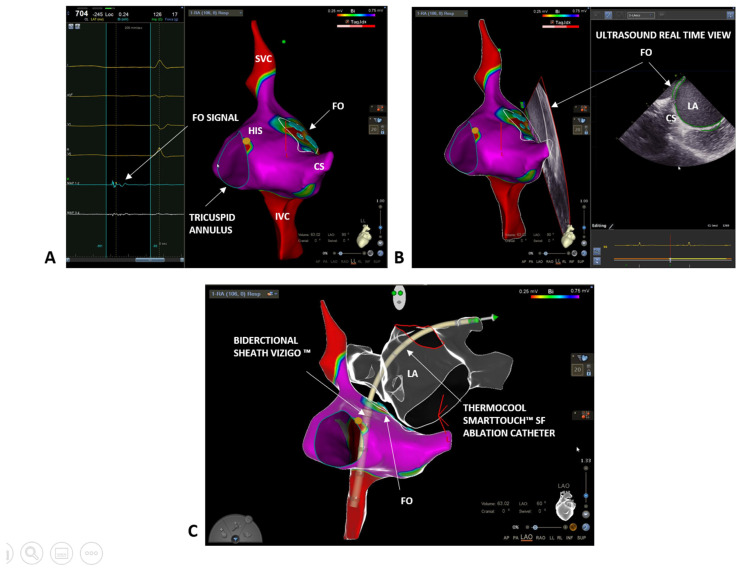
(**A**) Right atrium bipolar map (0.25–0.75 mV) with ThermoCool SmartTouch™ SF in a left lateral (LL) projection: blue tags indicate fragmented and low voltage signals, typical of the fossa ovalis (FO) area; yellow tags indicate His location. (**B**) Confirmation of FO location with ICE imaging and co-registration on the electro-anatomical map (LL projection) with CartoSound^®^ module. (**C**) Transseptal puncture with the bidirectional steerable sheath VIZIGO™ in a left anterior oblique (LAO) projection with the ablation catheter in the left superior pulmonary vein. Left atrium showed in glass mode. FO = fossa ovalis, LA = left atrium; SVC = superior vena cava; IVC = inferior vena cava; CS = coronary sinus.

We started the procedure by performing a bipolar map of the right atrium with a ThermoCool SmartTouch™ SF (Biosense Webster, Johnson & Johnson Medical S.p.a., Irvine, CA, USA) ablation catheter. First, we delineated the inferior and superior vena cava, and the coronary sinus, and we tagged in yellow the His potential (Figure 1A). Next, we carefully reconstructed the interatrial septum and, finally, to precisely define the fossa ovalis (FO), we set the color range on the bipolar map to 0.25–0.75 mV and we tagged in light blue all the fragmented and low voltage signals, which are typical of the FO area [13] (Figure 1A). The FO location was further confirmed by the ICE and the CartoSound^®^ module (Biosense Webster, Johnson & Johnson Medical S.p.a., Irvine, CA, USA). A 10F Soundstar™ Ultrasound catheter was advanced in the right atrium and pointed at the FO. Thanks to the CartoSound^®^ module, it was possible to mark the contours of the FO location and to visualize them on the 3D EAM previously obtained (Figure 1B). In addition to the FO, using ICE and the CartoSound^®^ module, it was possible to assess and to reconstruct the structures of the left atrium (left atrial appendage, pulmonary veins, and esophagus). Next, a single transseptal puncture was performed using the bidirectional guiding sheath VIZIGO™ (8.5F) and a transseptal needle (standard Brockenbrough needle 98 cm from Abbott, St. Paul, MN, USA). Since the sheath is visible on the Carto 3^®^ EAM system, it was possible to place it in the superior vena cava (3 to 4 cm above the cavoatrial junction) without any use of fluoroscopy. At this point, the guidewire was removed and the transseptal needle with the stylet was gently inserted and advanced through VIZIGO™, avoiding pushing the needle over the tip of the sheath. In this step, the needle and the sheath were held in the fingers between 4 and 6 o’clock. The needle and the sheath were pulled back caudally, visualizing the VIZIGO™ sheath with the 3D EAM system until it reached the level of the coronary sinus and the tip of the dilator inside the sheath placed in the FO. From this location, the VIZIGO™ sheath was pushed and placed on the FO under the guidance of the EAM system and confirmed by ICE imaging (Figure 1C). When the sheath was in the right position, the transseptal needle was advanced to perform the puncture. Live tracking of the distal part of the needle on the 3D EAM system was possible thanks to an Alligator clip lead wires (FIAB Spa, Florence, Italy) connected to the distal portion of the needle (see Video S1 in the Supplementary Materials). Both the transseptal sheath and the needle should be advanced for 1–2 cm in the left atrium (LA). Then, holding the needle, the sheath was advanced over the needle. Finally, the needle and the dilator were kept steady, and the sheath was advanced over the dilator. The transseptal sheath was deaired and a 0.032 inch guidewire was advanced toward the left superior pulmonary vein under ICE guidance.

After a successful transseptal puncture, the ThermoCool SmartTouch™ SF catheter was advanced through the VIZIGO™ sheath into the left atrium to perform a fast anatomical mapping and PVI following the CLOSE protocol [11]. With the help of the VIZIGO™ steerable sheath, we performed ablations with good contact and stability, leading to fast and effective PVI. There were no complications during the entire procedure. The procedure time was 100 min, radiofrequency time was 20 min, and, finally, the fluoroscopy time was 00:00 min:s. The patient was followed up 3 months after the procedure with an outpatient visit and electrocardiographic-Holter monitoring, which did not register any AF episode. After a 9 months follow-up, the patient presents no more symptoms, is not taking any antiarrhythmic drugs, and does not have recurrences of AF. He is taking apixaban 5 mg twice a day and ACE inhibitor drug for blood pressure control.

## 3. Case 2: Persistent Atrial Fibrillation

**Figure 2:**
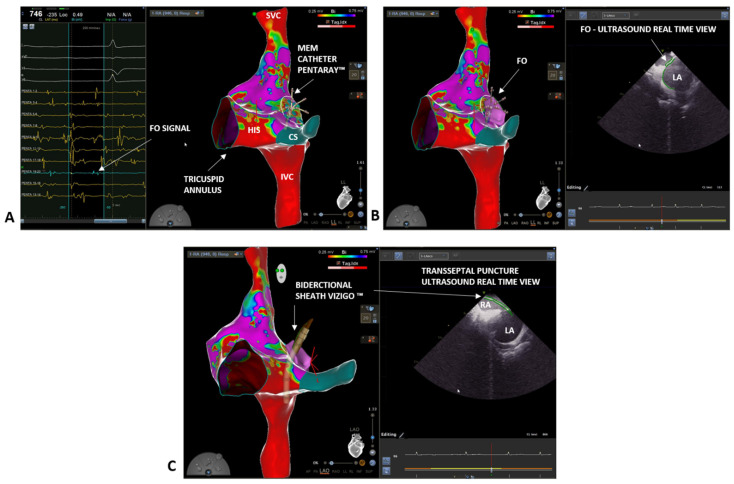
(**A**) Right atrium bipolar map (0.25–0.75 mV) (LL projection). Pentaray™ identification of the fragmented and low voltage signals, typical of the fossa ovalis (FO) area (blue tags). (**B**) Confirmation of FO location thanks to the ICE catheter and co-registration with the electro-anatomical map (LL projection) with CartoSound^®^ module. (**C**) Transseptal puncture with the VIZIGO™ steerable sheath, confirmed by ICE imaging. FO = fossa ovalis; SVC = superior vena cava; IVC = inferior vena cava; CS = coronary sinus.

**Figure 3:**
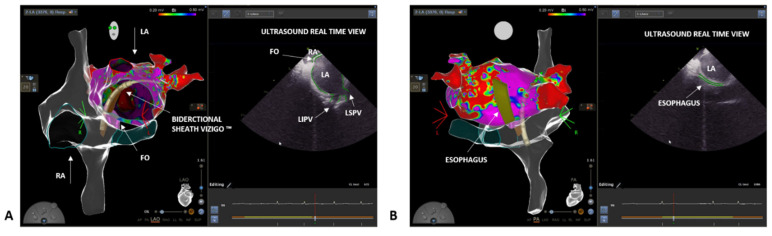
Detailed reconstruction of both right (glass mode) and left atrium (bipolar map) anatomy of the second case thanks to a combination of Carto 3^®^ EAM system, ICE, and CartoSound^®^ module in a LAO projection and in a postero-anterior projection. Accurate identification of pulmonary veins (**A**) and esophagus location (**B**) on ICE images and co-registration with the electro-anatomical maps using CartoSound^®^ module. FO = fosa ovalis; LA = left atrium; LIPV = left inferior pulmonary vein; LSPV = left superior pulmonary vein; RA = right atrium.

A 46-year-old male was referred to our clinic due to a long history of persistent atrial fibrillation. He had arterial hypertension and diabetes mellitus under good pharmacological control. He also had microcythemia due to a thalassemic trait. The atrial fibrillation was discovered seven years earlier with an electrocardiogram performed during a routine medical examination. The patient was asymptomatic for palpitations, irregular heartbeat, or other cardiological symptoms. An echocardiogram was performed without showing any pathological feature and oral anticoagulation with Dabigatran (CHA2DS2-VASC score 2) was started. After four weeks of therapy, electrical cardioversion was performed with effectiveness in restoring sinus rhythm. An antiarrhythmic therapy with Flecainide 100 mg twice daily was also initiated. The patient did not undergo further medical checks during the following five years, when, during a cardiological visit, the recurrence of the arrhythmia was discovered. The patient mentioned reduction in daily normal activity due to asthenia. An echocardiography exam showed a left atrial indexed volume of 36 mL/m2 and a left ventricular ejection fraction of 47%. AF ablation through PVI was proposed. At admission, the electrocardiogram showed AF. To perform an accurate anatomical reconstruction of the right and left atrium and a high-density voltage map, the multi-electrode mapping (MEM) catheter Pentaray™ (Biosense Webster, Johnson & Johnson Medical S.p.a., Irvine, CA, USA) was used. As in the previous case, we started by mapping the right atrium to define the inferior and superior vena cava, the His location, and the coronary sinus (Figure 2A). Next, a decapolar catheter was inserted in the coronary sinus under 3D EAM guidance. Again, we mapped the FO looking for fragmented and low-voltage signals. However, this time, using the Pentaray™ catheter, we were able to achieve a higher signal resolution and to easily discriminate between a low-voltage and fragmented signal area, although the patient was in AF. Moreover, using the Pentaray™ catheter, we were able to acquire a detailed map in a shorter time (5 min with Pentaray™ catheter vs. 10 min with Thermocool SmartTouch™ SF catheter in the previous case). Again, based on the 3D EAM and under ICE guidance, a single transseptal puncture was performed with the VIZIGO™ sheath and the transseptal needle, both visible on the 3D EAM system (Figure 2B,C). The transseptal puncture procedure followed the same steps already described in the previous case (video of the puncture is available in the Supplementary Materials). The Pentaray™ catheter was then advanced in the left atrium through the VIZIGO™ sheath and a detailed fast anatomical mapping of the chamber and the veins was obtained (Figure 3). The VIZIGO™ steerable sheath was extremely useful to map all the left atrium structures and veins very quickly. Moreover, using ICE imaging and the CartoSound^®^ module, we could identify important structures, such as the pulmonary veins (Figure 3A) and the esophagus (Figure 3B), and to co-register the ultrasound images with the fast anatomical map obtained with CARTO 3^®^. The Pentaray™ catheter was then replaced by the ThermoCool SmartTouch™ SF catheter to perform PVI following the CLOSE protocol [14]. Again, with the help of the VIZIGO™ steerable sheath, we performed ablations with good contact and stability, leading to a fast and effective PVI. As in the previous case, the entire procedure was performed without fluoroscopy and without complications (procedural time was 110 min, radiofrequency time was 19 min, and fluoroscopy time was 00:00 min:s). The patient was followed up 3 months after the procedure with an outpatient visit, echocardiogram, and electrocardiographic-Holter monitoring, which did not register any AF episode. The echocardiogram showed a normal left ventricular ejection fraction (65%) and the patient mentioned that there was not a reduction in normal daily activities. After 6 months follow-up, the patient is taking low dose of flecainide (50 mg twice per day), apixaban 5 mg twice per day and has not recurrences of AF.

## 4. Discussion

The first experience that highlighted the possibility of reducing fluoroscopy time by mapping systems was published in 2000 [15]. Since then, data supporting this innovative approach was collected and published. Year by year, the so-called near zero and complete zero-fluoroscopy techniques has aroused interest and has gained international approval even among the most skeptical physicians. To date, the majority of data collected are supporting this approach in right-sided arrhythmias, such as cavotricuspid isthmus dependent flutter, atrioventricular reentrant tachycardia, and atrioventricular nodal reentry tachycardia [16].

These two case reports show that complete zero fluoroscopy is feasible in paroxysmal and persistent atrial fibrillation, both mapping in sinus rhythm and in AF [17]. The major hurdle is to reach zero fluoroscopy during a transseptal puncture maneuver. The transseptal puncture is an old technique first described in the late 1950s [18,19,20,21]. Despite the increase in AF procedures over the years, the transseptal technique has mostly remained the same. Therefore, the need for precise and safe punctures in elective procedures, such as AF ablation, has stimulated novel approaches and devices. One of the safest features added was the introduction of echocardiography that can be transesophageal or intracardiac. Recently, Yu and colleagues proposed a workflow that included the possibility to see the transseptal needle with 3D EAM, but to visualize the needle, the needle needs to be exposed during the pullback [22]. We have presented a novel workflow that allows to perform a completely zero-fluoroscopy ablation procedure including the transseptal puncture phase, minimizing the risks of complications (see Video on zero-fluoroscopy transeptal puncture workflow). We combined an accurate reconstruction of the interested structures with the CARTO 3^®^ EAM system and the ICE imaging combined with the CartoSound^®^ Module. The VIZIGO™ steerable guiding sheath is visible with the 3D EAM system and we can maneuver under 3D EAM visualization. This allows to expose and visualize the distal part of the needle only when the dilator of the sheath is tenting the FO without risk.

Although the use of ICE may increase the cost of the overall procedure, it comes with the great advantage of increased safety and a reduced risk of complications [23]. Moreover, ICE allows to image the left atrium from the right atrium providing useful information on possible anatomical abnormalities, hence giving the operator the opportunity to optimize the transseptal puncture for optimal maneuverability. Furthermore, through ICE and CartoSound^®^, we can delineate the location of crucial structures, such as the esophagus. Finally, thanks to ICE imaging, we can constantly monitor pericardial effusion formation or thrombus formation in the left atrium. The VIZIGO™ steerable sheath has, additionally, several advantages since it is visible on the CARTO 3^®^ EAM system, abolishing the need for fluoroscopy. Moreover, it increases contact force and stability during RF ablation.

## 5. Conclusions

In summary, the combination of Carto 3^®^ EAM system, ICE, and CartoSound^®^ module, together with the use of the VIZIGO™ steerable sheath, enables completely zero-fluoroscopy procedures, without compromising the safety of the procedure. Whether better contact force and improved stability result in faster pulmonary vein isolation and increase efficacy and efficiency needs to be proved by further studies.

## Data Availability

Source data files are available upon request.

## Supplementary Materials

The following are available online at https://www.mdpi.com/article/10.3390/biology10121333/s1, Video S1: Video on zero-fluoroscopy transeptal puncture workflow.
